# Geographic and Socioeconomic Disparities in Emergency Presentations Among Colorectal Cancer Patients in Victoria, Australia

**DOI:** 10.1002/cam4.70909

**Published:** 2025-05-04

**Authors:** Bedasa Taye Merga, Nikki McCaffrey, Suzanne Robinson, Craig Sinclair, Justin M. Yeung, Anita Lal

**Affiliations:** ^1^ School of Public Health, College of Health and Medical Sciences Haramaya University Harar Oromia Ethiopia; ^2^ Deakin Health Economics, School of Health and Social Development Deakin University Melbourne Victoria Australia; ^3^ Cancer Council Victoria East Melbourne Victoria Australia; ^4^ Western Precinct, The University of Melbourne Melbourne Victoria Australia; ^5^ Department of Colorectal Surgery Western Health Melbourne Victoria Australia

**Keywords:** cancer, colorectal, disparity, emergency presentation, inequality

## Abstract

**Background:**

Disparities in cancer care access and utilisation influence the stage of diagnosis and pathways of care. Colorectal cancer (CRC) patients presenting as emergencies often have advanced disease and poorer outcomes. This study aimed to assess geographic and socioeconomic disparities in emergency presentations (EPs) of CRC patients in Victoria, Australia.

**Methods:**

Linked datasets from a Victorian population‐based cancer registry and emergency and hospital admissions were analysed for CRC patients diagnosed between 2009 and 2022. Concentration indices (CIs) assessed the distribution of EPs by socioeconomic position and remoteness. Multivariable logistic regression identified factors associated with EPs, with results presented as adjusted odds ratios and 95% confidence intervals. In all analyses, statistical significance was determined using a *p*‐value threshold < 0.05.

**Results:**

A total of 24,236 CRC patients had emergency department (ED) visits for any reason. Twenty‐one per cent (5086) of them reported CRC‐related symptoms. Among these, 33.8% (1721) presented within 6 months before diagnosis. The concentration indices indicated that EPs were disproportionately higher among the most disadvantaged quintiles (CI = −0.060, *p*‐value < 0.001) and regional and remote areas (CI = −0.065, *p*‐value < 0.001). Multivariable logistic regression showed higher odds of EPs among socioeconomically disadvantaged groups (Q1: AOR = 1.25; Q2: AOR = 1.31) compared to the least disadvantaged (Q5). Similarly, patients in regional and remote areas had higher odds of EP than those in major cities (inner regional: AOR = 1.26; outer regional/remote: AOR = 1.52). Advanced‐stage diagnoses compared to early stages (stage 4: AOR = 1.67), whereas older age groups had lower odds compared to 45–49 age groups (65–69 years: AOR = 0.67, > = 75 years, AOR = 0.60 to 0.70).

**Conclusions:**

Enhancing access to primary care and strengthening cancer screening programs, particularly in socioeconomically disadvantaged and regional, and remote communities, could reduce disparities, promote earlier diagnosis, and improve outcomes. Prioritising targeted interventions in these populations is essential to addressing these inequities.

## 1 Introduction

Colorectal cancer (CRC) ranks as the third most commonly diagnosed cancer worldwide and the second leading cause of cancer‐related mortality, with 1.9 million new cases and 930,000 deaths reported globally in 2022 [1]. In Australia, 15,542 new cases of colorectal cancer were diagnosed in 2024, and approximately 5239 colorectal cancer‐related deaths were recorded [2]. CRC is largely preventable and treatable if identified early through effective screening and timely detection [3]. While early detection can significantly improve outcomes, disparities in healthcare access and utilisation can lead to differences in the stage at diagnosis and the pathways through which patients present for care [4, 5].

The National Bowel Cancer Screening Program (NBCSP) in Australia offers free biennial screening for individuals aged 45–74 to improve early colorectal cancer (CRC) detection [6, 7]. Participation in the NBCSP has been shown to reduce advanced‐stage CRC diagnoses and lower rates of emergency presentations (EPs), highlighting its potential to improve patient outcomes [8, 9]. However, a significant proportion of CRC patients still present as emergencies, often indicating delayed detection and advanced disease [10]. EPs are associated with poorer clinical outcomes and impose a substantial healthcare burden, as these patients require longer hospital stays and incur higher treatment costs compared to those diagnosed through non‐emergency pathways [11]. The rate of EP among patients newly diagnosed with CRC (within 6–12 months of diagnosis) serves as a critical quality indicator, highlighting missed opportunities for early detection and timely intervention that could improve patient outcomes [5, 12, 13, 14, 15]. Globally, studies indicate that between 14% and 33% of colorectal cancers are diagnosed as emergencies [16, 17]. In Australia, one in five newly diagnosed cancer patients present to ED 6 months prior to cancer diagnosis, and one in ten is diagnosed as a result of their ED visit [18].

Geographic and socioeconomic factors are critical determinants of health that influence access to healthcare services and cancer outcomes [19, 20]. Socioeconomic factors encompass various dimensions, including income, education, employment, and living conditions, which can impact an individual's ability to seek and receive timely health care [21]. In Australia, approximately 28% of the population resides in regional and remote areas, where barriers such as long travel distances, limited availability of services, and transportation challenges can delay CRC diagnosis and treatment [22, 23, 24]. These barriers increase the risk of EPs, where patients are more likely to present with advanced disease and face poorer clinical outcomes compared to those presenting through non‐emergency pathways [4, 5].

Although the impact of socioeconomic and geographic factors on health disparities is well‐established, their influence on EPs among CRC patients in Victoria has not been fully analysed [25]. Understanding these disparities is critical to inform targeted interventions and healthcare planning that can reduce EPs and improve outcomes for disadvantaged populations. Therefore, this study aims to examine geographic and socioeconomic disparities in EPs among CRC patients in Victoria.

## 2 Methods

### 2.1 Data Sources

The study utilised Victorian Cancer Registry (VCR) data for CRC patients from 1 January 2009 to 31 December 2022 linked to the Victorian Emergency Minimum Datasets (VEMD) and Victorian Admission Episodes Datasets (VAED). The VCR is a comprehensive cancer registry for Victoria state, collecting cancer data from 254 hospitals, 11 radiotherapy centres, 7 interstate registries, screening registries, 26 pathology laboratories, and death registries [26]. The VCR dataset includes demographic and socioeconomic variables and cancer site and stage of cancer. The CRC patients were identified using topography codes C18 (colon) and C19–C20 (rectal) from the International Classification of Diseases, Tenth Revision (ICD‐10). The VEMD datasets contain admission/arrival and discharge dates and costs.

### 2.2 Outcome Variables

The EP for CRC‐related symptoms within 6 months before the cancer diagnosis was the main outcome variable. Furthermore, the number of EP within 30 days of hospital admission and their variations by remoteness areas, socioeconomic quintiles, and stage at diagnosis were estimated.

### 2.3 EPs 6 Months Before Cancer Diagnosis

EPs involving CRC‐related symptoms, such as changes in bowel habits, rectal bleeding or blood in stool, diarrhoea, constipation, abdominal pain, weight loss, and anaemia, were included. These symptoms are identified by the Centers for Disease Control and Prevention (CDC) [27] and Cancer Australia [28] to be relevant to CRC. EP within 6 months before cancer diagnosis was derived from the difference between the cancer diagnosis date and the ED arrival date. Patients were coded as 1 if they had at least one emergency visit within 6 months before diagnosis, and 0 otherwise. For multiple visits within 6 months, the latest ED visit before diagnosis was used.

### 2.4 Emergency Presentations Within 30 Days of Treatment or Procedure for CRC

CRC patients who had records of hospital admissions and ED following cancer diagnosis were included. Hospital admissions due to colorectal resection, major small and large bowel procedures, chemotherapy, radiotherapy, and colonoscopy were considered. Reasons for hospital admissions unrelated to the CRC were excluded based on the diagnostic‐related group (DRG) codings [29]. The advice of a colorectal surgeon was sought to validate DRG codes and treatments related to CRC.

### 2.5 Independent Variables

Independent variables used in this study were selected based on previous studies [9, 25, 30, 31, 32], and included a range of demographic, socioeconomic, and cancer‐related factors. Explanatory variables.

The main explanatory variables were socioeconomic position and remoteness of areas.

Socioeconomic position is assigned based on the Socio‐Economic Indexes for Areas (SEIFA), Index of Relative Socioeconomic Disadvantage (IRSD), summarising variables that indicate relative disadvantage [21]. The SEIFA index categorises individuals into groups residing in similarly ranked areas based on information such as residents' income, qualifications, and occupational skills [21]. The SEIFA scores were categorised into five equal groups (quintiles 1–5). Quintile 1 referred to the most socioeconomically disadvantaged areas.

Remoteness was categorised according to the Accessibility/Remoteness Index of Australia (ARIA) measures relative geographic access to services. The ARIA groups areas as major cities, inner regional, outer regional, remote, and very remote areas [23]. Patients' residential postcodes were used to assign remoteness categories based on ARIA classifications. There are no very remote areas in Victoria, and since the remote category had very few observations (< 0.10%), these were merged with the outer regional area for better model predictions.

### 2.6 Covariates

Age at diagnosis was divided into age groups: 45–49, 50–54, 55–59, 60–64, 65–69, 70–74, 75–79, 80–84, and 85+ years. Gender was categorized as male or female. Stage at diagnosis was classified into the four stages of CRC classified according to the Australian Clinico‐pathological Staging (ACPS) system from A to D, indicating how far the cancer has spread anatomically. Cancer site was classified into colon cancer and rectal cancer [33].

Country of birth was categorized based on the language spoken, classifying countries as English‐speaking and non‐English‐speaking countries.

### 2.7 Data Analysis

STATA version 16.1 (StataCorp, College Station, TX, USA) was used for data analysis. Descriptive statistics described CRC patients registered between 2009 and 2022. The distribution of EPs by socioeconomic positions, remoteness areas, and other covariates were presented by proportions and compared using chi‐square. The length of hospital stays was estimated as the difference between admission and discharge dates. Median costs of ED visits were estimated and compared among remoteness areas and SEIFA quintiles. The Kruskal‐Wallis test was used to test the statistical significance of variations in the median costs of ED visits among socioeconomic groups and remoteness areas [34]. Logistic regression models were used to examine the associations between EP and explanatory variables and covariates. The analysis followed a two‐step approach. First, each variable was entered into a bi‐variable logistic regression model. Second, variables that were significant at a *p*‐value of less than or equal to 0.25 in bi‐variable analysis were fitted into a multivariable logistic regression model to adjust the associations between EP and independent variables. Socioeconomic position (SEIFA), remoteness areas (ARIA), age at diagnosis, country of birth, stage at diagnosis, gender, and cancer site were fitted in the multivariable analysis. The associations were presented with adjusted odds ratios and 95% confidence intervals. In all analyses, statistical significance was determined using a *p*‐value < 0.05. The model adequacy was tested by Hosmer and Lemeshow's test of goodness of fit [35].

### 2.8 Concentration Curve and Concentration Index

The concentration index (CI) was used to quantify the degree of socioeconomic and geographic disparities in EPs among CRC patients. Since its introduction by Wagstaff et al. [36] in 1991, the concentration index has become a standard measure for quantifying socioeconomic‐related inequalities in health. It has been extensively used across a wide range of studies to assess disparities in health outcomes such as mortality rates, disease prevalence, and access to or use of healthcare services [37, 38]. The CI is valued for its ability to capture both the direction and magnitude of inequality, allowing researchers and policymakers to identify whether health outcomes are disproportionately concentrated among the poor or the rich [39]. The CI is defined as: [40]

$$\mathrm{CI}=\frac{2}{\mu }\mathrm{cov}\left(\mathrm{yi},\mathrm{Ri}\right)$$

where CI represents the concentration index, yi denotes the EP (EP), μ is the mean EP, and *Ri* is the cumulative percentage that each participant represents over the total population, categorised by the distribution of SEIFA and AIRA, $\mathrm{cov}\left(\mathrm{yi},\mathrm{Ri}\right)$ is the covariance between yiandRi. Since the outcome variable in this study is binary (either EP or no EP), the limits of the CI depend on the mean value of the outcome variable and do not range from −1 to 1. Consequently, the CI ranges from μ − 1 (lower limit) to 1 − μ (upper limit). To address this limitation, we used the Erreygers normalised concentration index $\left(\mathrm{ECI}\right),$ a modified version of the general concentration index: [41]

$$\mathrm{ECI}=4*\mu *\mathrm{CI}\left(y\right)$$

Socioeconomic and geographic disparities in EP were visualised using a concentration curve [42]. The curve illustrates the cumulative percentage of EP on the y‐axis against the cumulative percentage of CRC patients ranked by SEIFA quintiles and AIRA groups on the x‐axis, sorted from Q1 to Q5 and from remote areas to major cities, respectively. The direction of the association between EP, SEIFA, and AIRA disparities is indicated by the line of equality (at 45°). The curve falling below the line of equality indicates a positive association (EP is concentrated among Q5 or major cities), while a curve above the line of equality indicates a negative association (EP is prevalent among Q1 or remote areas) [39]. When there are no socioeconomic and geographic disparities (perfect equality), the concentration index is zero, and the concentration curve lies directly on the line of equality at 45°.

## 3 Results

### 3.1 Study Participants

A total of 24,236 CRC patients diagnosed in Victoria between June 2009 and June 2022 with records of ED visits were identified for analysis. Almost half (51.1%) were male, 25.7% were from the most disadvantaged quintile (quintile 1), 72% were from English‐speaking countries, 66.2% were from major cities, and only 0.10% were from remote areas (Table 1).

**TABLE 1:** Characteristics of study participants.

Variables	Categories	Frequency (%)
Gender	Male	12,393 (51.1)
	Female	11,843 (48.9)
SEIFA Quintiles	Q1	6182 (25.7)
	Q2	5367 (22.3)
	Q3	4835 (20.1)
	Q4	4239 (17.6)
	Q5	3450 (14.3)
Country of birth by language	English speaking	17,443 (72)
	Non‐English speaking	6793 (28)
ARIA groups	Major cities	16,035 (66.2)
	Inner regional	6271 (25.9)
	Outer regional	1906 (7.9)
	Remote	24 (0.10)
Age at cancer diagnosis (years)	45–49	803 (3.3)
	50–54	1295 (5.3)
	55–59	1683 (6.9)
	60–64	2347 (9.7)
	65–69	3010 (12.4)
	70–74	3692 (15.2)
	75–79	3692 (15.2)
	80–84	3795 (15.7)
	85+	3634 (15.0)
Stage at diagnosis	Stage 1	4983 (20.6)
	Stage 2	6715 (27.7)
	Stage 3	5740 (23.7)
	Stage 4	3849 (15.9)
	Unknown	2949 (12.2)
Sites of cancer	Colon	23,765 (98.1)
	Rectum	471 (1.9)

Abbreviations: ARIA, accessibility/remoteness index of Australia; SEIFA, socioeconomic index for areas.

Fifteen per cent of CRC patients were diagnosed in the 80–84 years age group, while only 3.3% were diagnosed 45–49 years of age. Most patients were diagnosed at stage 2 of the disease (27.7%). Almost all (98.1%) patients were diagnosed with colon cancer rather than rectal cancer (Table 1).

### 3.2 EPs 6 Months Before CRC Diagnosis

**FIGURE 1:**
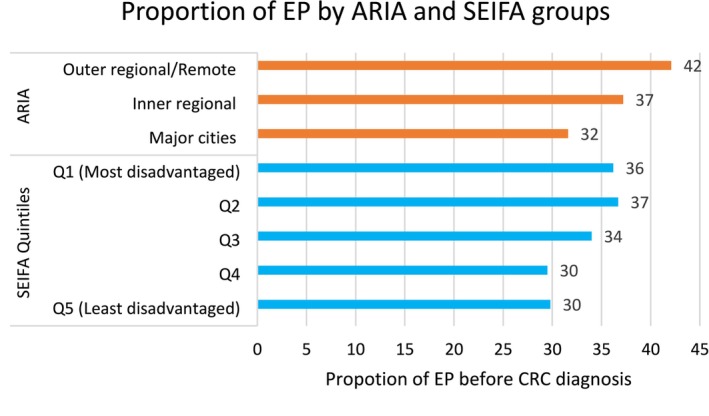
Distributions of EPs in 6 months before diagnosis by SEIFA and ARIA groups.

Twenty‐nine per cent (6975) of CRC patients had EPs 6 months before their cancer diagnosis for any reason. Twenty‐one per cent (5086) of ED presenters had CRC‐related symptoms, with 33.8% (1721) of these cases occurring within 6 months before diagnosis. Patients from the most disadvantaged quintiles (Q1 and Q2) had the highest proportions of EPs (36%–37%), whereas those in the least disadvantaged quintile (Q5) had the lowest (30%) (Figure 1, blue bars). Similarly, geographic disparities were evident, with EP rates increasing from 32% in major cities to 42% in outer regional and remote areas (Figure 1, orange bars).

Both females and males have almost similar proportions of EPs, (33.6% vs. 34.1% respectively). The proportion of EPs was highest among the 50–54, 60–64, and 65–69 age groups, with 40.7%, 38.1%, and 40.2% respectively, and for patients diagnosed at Stage 4 (41.7%) (Table 2).

**TABLE 2:** EPs due to CRC‐related symptoms by patient characteristics.

Variables	Categories	EP 6 months before cancer diagnosis frequency (%)		Chi‐square *p*
		No	Yes	
Total (*n* = 5086))		3365 (66.2)	1721 (33.8)	
SEIFA Quintiles	Q1	867 (63.8)	492 (36.2)	*χ* ^2^ = 19.60 *p*‐value < 0.01
	Q2	736 (63.3)	426 (36.7)	
	Q3	669 (66)	344 (34)	
	Q4	622 (70.5)	260 (29.5)	
	Q5	447 (70.2)	190 (29.8)	
ARIA	Major cities	2325 (68.4)	1072 (31.6)	*χ* ^2^ = 27.20 *p*‐value < 0.01
	Inner regional	794 (62.8)	470 (37.2)	
	Outer regional/remote	246 (57.9)	179 (42.1)	
Gender	Male	1626 (66.4)	822 (33.6)	*χ* ^2^ = 0.14 *p*‐value = 0.71
	Female	1739 (65.9)	899 (34.1)	
Country of birth by language	English speaking	2368 (65.8))	1230 (34.2)	*χ* ^2^ = 0.66 *p*‐value < 0.41
	Non‐english speaking	997 (67)	491 (33)	
Age at diagnosis	45–49	537 (66.9)	266 (33.1)	*χ* ^2^ = 27.70 *p*‐value < 0.01
	50–54	124 (59.3)	85 (40.7)	
	55–59	194 (66)	100 (34)	
	60–64	241 (61.9)	148 (38.1)	
	65–69	320 (59.8)	215 (40.2)	
	70–74	404 (69.2)	180 (30.8)	
	75–79	452 (65.6)	237 (34.4)	
	80–84	549 (67.6)	263 (32.4)	
	85+	532 (66.7)	266 (32.3)	
Stage at diagnosis	Stage 1	469 (70.5)	196 (29.5)	*χ* ^2^ = 42.90 *p*‐value < 0.01
	Stage 2	927 (69.7)	403 (30.3)	
	Stage 3	883 (65.8)	459 (34.2)	
	Stage 4	657 (58.3)	469 (41.7)	
	Unknown stage	429 (68.9)	194 (31.1)	
Cancer site	Colon	3307 (66.2)	1685 (33.8)	*χ* ^2^ = 0.85 *p*‐value = 0.36
	Rectum	58 (61.7)	36 (38.3)	

Abbreviations: ARIA, accessibility/remoteness index of Australia; EP, emergency presentations; SEIFA, socioeconomic index for areas.

### 3.3 Trends of EPs 6 Months Before Cancer Diagnosis

**FIGURE 2:**
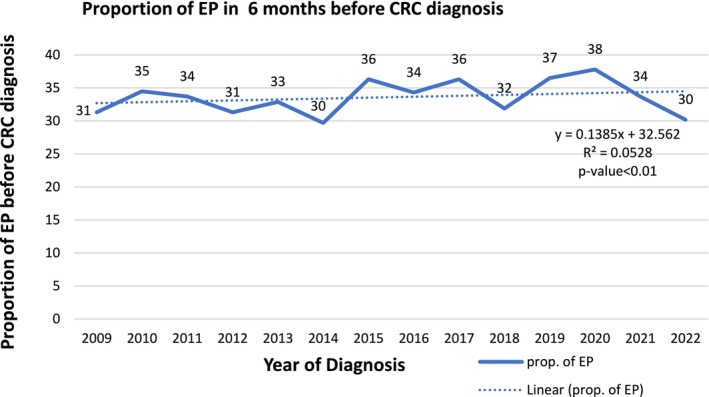
Trends of EP 6 months before cancer diagnosis.

The proportion of EPs 6 months before diagnosis for CRC‐related symptoms fluctuated over the years, with notable peaks and troughs (Figure 2). The highest proportion is observed in 2020 (38%), and the lowest in 2014 and 2022 (30%). A significant increase occurred in 2019 (37%) and peaked in 2020 at 38%. After 2020, there is a sharp decline, with 2021 dropping to 34% and 2022 further down to 38%.

### 3.4 Concentration Index and Curve for Socioeconomic and Geographic Inequalities in EP

**FIGURE 3:**
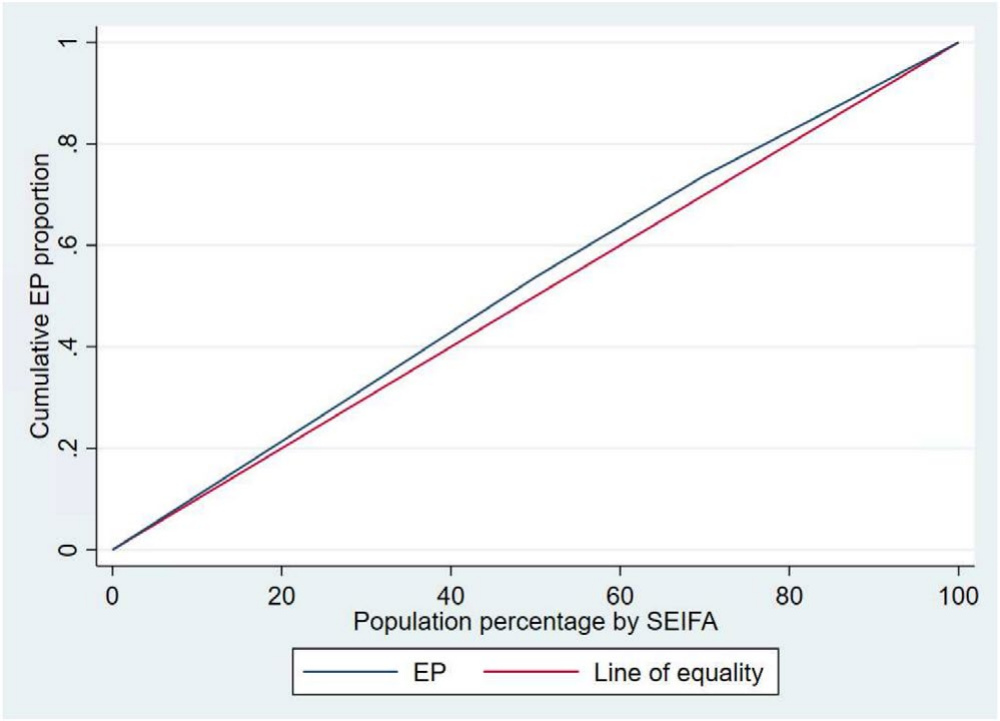
Concentration curve of EPs by socioeconomic positions.

**FIGURE 4:**
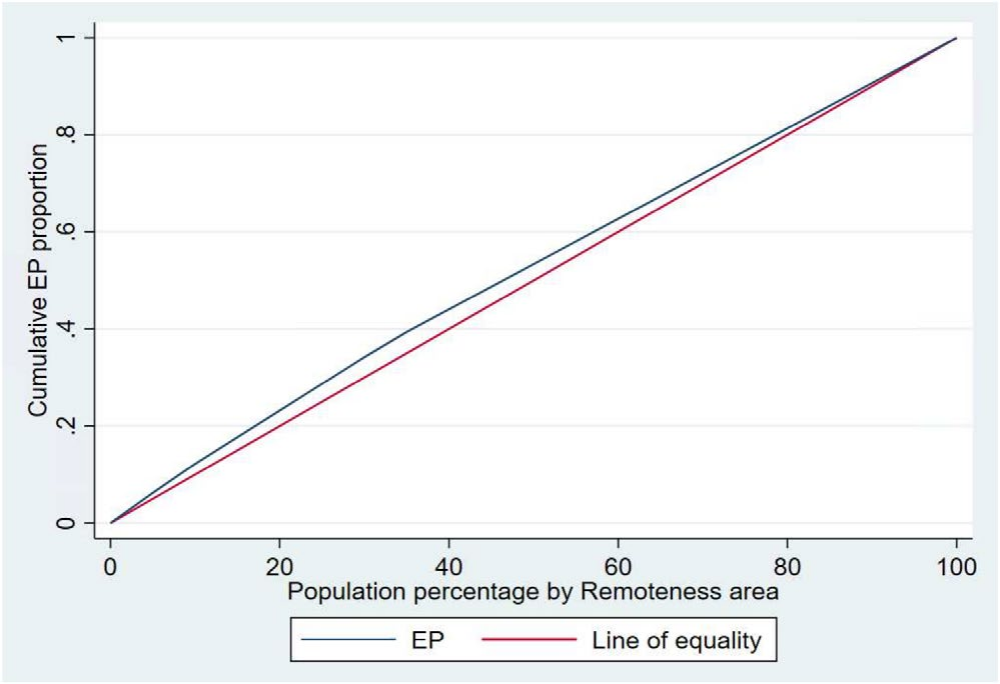
Concentration curve of EP by remoteness areas.

The Concentration index (CI) values of −0.0596, (*p*‐value < 0.001) for socioeconomic position and CI = −0.0650 for regional and remote areas (*p*‐value < 0.001) indicate EPs are slightly more concentrated among disadvantaged groups. Concentration curves show that the line of the EP lies above the line of equality, demonstrating that EPs were disproportionately accumulated among the disadvantaged groups. The area between the equality line and the EP curve indicates the extent of inequality; the wider the area, the higher the inequality (Figures 3 and 4).

### 3.5 Length of ED Stays and Costs

More than three‐quarters of CRC patients who visited the ED stayed for less than 24 h (same‐day admission), 23.4% stayed for 24–48 h, and 0.02% stayed for more than 48 h.

The median total cost was AUD 762 (IQR AUD 936). The costs varied across different socioeconomic quintiles and remoteness areas. Quintile 2 had the lowest median total cost at AUD 752, and quintile 5 had the highest (AUD 789). Outer regional and remote areas had the lowest median total cost at AUD 644, and major cities had the highest at AUD 816 (Table 3).

**TABLE 3:** Length of ED stays and costs.

Variables	Categories	The proportion of the length of ED stays		
		Same‐day admission, *n* (%)	1 day, *n* (%)	2 days, *n* (%)
*N* = 24,073		18,425 (76.5)	5643 (23.4)	5 (0.02)
SEIFA	Q1	4642 (75.1)	1538 (24.9)	2 (0.03)
	Q2	4116 (76.7)	1251 (23.3)	0
	Q3	3712 (76.8)	1122 (23.2)	1 (0.02)
	Q4	3296 (77.8)	942 (22.2)	1 (0.02)
	Q5	2659 (77.1)	790 (22.9)	1 (0.03)
	Chi‐square	*χ* ^2^ = 13.1, *p*‐value = 0.11		
ARIA groups	Major cities	12,149 (75.8)	3882 (24.2)	4 (0.02)
	Inner regional	4835 (77.1)	1435 (22.9)	1 (0.01)
	Outer regional/Remote	1566 (81.1)	364 (18.9)	0
	Chi‐square	*χ* ^2^ = 29.6, *p*‐value = 0.00		

Median (interquartile ranges) cost of ED by SEIFA quintiles and ARIA groups				
Variables	Categories	Cost categories		
		Indirect cost	Direct cost	Total cost
*N* = 16,700		$144 (153)	$609 (796)	$762 (936)
SEIFA	Q1	$147 (153)	$597 (764)	$755 (902)
	Q2	$144 (147)	$595 (730)	$751 (870)
	Q3	$140 (147)	$612 (781)	$758 (926)
	Q4	$142 (157)	$629 (838)	$777 (975)
	Q5	$15 (166)	$642 (935)	$789 (1097)
		*χ* ^2^ = 7.89 *p*‐value = 0.09	*χ* ^2^ = 4.04 *p*‐value = 0.40	*χ* ^2^ = 2.69 *p*‐value = 0.61
ARIA groups	Major cities	$148 (180)	$662 (939)	$816 (1121)
	Inner regional	$140 (113)	$555 (590)	$705 (672)
	Outer regional/Remote	$123 (109)	$504 (583)	$644 (656)
	Kruskal–Wallis test	*χ* ^2^ = 46.7 *p*‐value = 0.00	*χ* ^2^ = 88.5 *p*‐value = 0.00	*χ* ^2^ = 77.5 *p*‐value = 0.00

Abbreviations: ARIA, accessibility/remoteness index of AustraliaED, emergency department; SEIFA, socioeconomic index for areas.

In multivariable logistic regressions out of seven variables fitted, four variables: SEIFA quintiles, AIRA groups, age at diagnosis, and stage at diagnosis had statistically significant associations with EP within 6 months before cancer diagnosis. Compared to quintile 5 (least disadvantaged), quintiles 1 and 2 had higher odds of presenting at the ED before cancer diagnosis (Q1: AOR = 1.25; Q2 = 1.31). Patients from inner regional areas were 1.26 times more likely to present to the ED compared to those from major cities, and patients from outer regional/remote areas had 1.52 times higher odds of presenting to the ED compared to those from major cities.

Age at diagnosis showed statistically significant associations with EPs. Compared to age groups 45–49, those diagnosed at 65–69 and > =75 years had lower odds of presenting to ED (65–69 years: AOR = 0.67, > = 75 years, AOR = 0.60–0.70).

The odds of ED presentations with stage 4 cancer at diagnosis were 1.67 times higher compared to those who were diagnosed with stage 1 (Table 4).

**TABLE 4:** Factors associated with EPs 6 months before cancer diagnosis.

Variables	Categories	EP 6 months before cancer diagnosis	
		AOR (95% CI)	*p*
SEIFA Quintiles	Q 1	1.25 (1.01–1.54)	0.04
	Q 2	1.31 (1.05–1.62)	0.02
	Q 3	1.14 (0.91–1.42)	0.25
	Q 4	0.97 (0.77–1.22)	0.80
	Q 5	Reference	
ARIA groups	Major cities	Reference	
	Inner regional	1.26 (1.08–1.46)	0.00
	Outer regional/remote	1.52 (1.22–1.90)	0.00
Country of birth by language	English speaking	Reference	
	Non‐English speaking	1.06 (0.93–1.20)	0.41
Age at diagnosis (years)	45–49	Reference	
	50–54	0.77 (0.52–1.12)	0.17
	55–59	0.90 (0.63–1.28)	0.56
	60–64	0.95 (0.68–1.33)	0.77
	65–69	0.67 (0.48–0.94)	0.02
	70–74	0.74 (0.53–1.04)	0.08
	75–79	0.67 (0.48–0.93)	0.02
	80–84	0.70 (0.50–0.97)	0.03
	85+ years	0.60 (0.43–0.83)	0.00
Stage at diagnosis	Stage 1	Reference	
	Stage 2	1.06 (0.86–1.31)	0.57
	Stage 3	1.21 (0.99–1.50)	0.07
	Stage 4	1.67 (1.35–2.07)	0.00
	Unknown stage	1.08 (0.85–1.39)	0.52
Cancer site	Colon	0.79 (0.52–1.22)	0.30
	Rectum	Reference	
Gender	Male	Reference	
	Female	1.03 (0.92–1.17)	0.57

Abbreviations: AOR, adjusted odds ratio; ARIA, accessibility/remoteness index of Australia; CI, confidence interval; EP, emergency presentation; SEIFA, socioeconomic index for areas.

### 3.6 EPs Within 30 Days After a Hospital Admission

A total of 10,182 hospital admissions related to CRC with ED records were identified. Of these, 3771 admissions who had ED presentations following hospital admissions were included in the analysis. Out of 3771 hospital admissions with EP following the admission, 197 (5.2%) had EPs within 30 days of hospital admission. There were no statistically significant differences in EPs within 30 days of hospital admission across different remoteness areas, socioeconomic quintiles, regions of birth, gender, age groups, cancer types, and cancer stages (Table 5).

**TABLE 5:** Patient characteristics of CRC patients who had EPs within 30 days of hospital admission.

Variables	Categories	EP within 30 days of hospital admissions		Chi‐square (*χ* ^2^) *p*
		No frequency (percent)	Yes frequency (percent)	
Total (*n* = 3771)		3574 (94.8)	197 (5.2%)	
ARIA groups	Major cities	2455 (95.3)	121 (4.7)	*χ* ^2^ = 5.35 *p*‐value = 0.07
	Inner regional	884 (93.4)	63 (6.6)	
	Outer regional/remote	235 (94.8)	13 (5.2)	
SEIFA quintiles	Q 1	838 (94.5)	49 (5.5)	*χ* ^2^ = 4.72 *p*‐value = 0.32
	Q 2	736 (94.4)	44 (5.6)	
	Q 3	686 (94.8)	445 (6.2)	
	Q 4	675 (96.2)	27 (3.8)	
	Q 5	623 (95.3)	31 (4.7)	
Country of birth by language spoken	English speaking	2648 (94.9)	142 (5.1)	*χ* ^2^ = 0.038 *p*‐value = 0.85
	Non‐English speaking	871 (94.7)	49 (5.3)	
Gender	Male	1901 (94.4)	113 (5.6)	*χ* ^2^ = 1.30 *p*‐value = 0.25
	Female	1673 (95.2)	84 (4.8)	
Age (years)	45–49	112 (91.1)	11 (8.9)	*χ* ^2^ = 10.4 *p*‐value = 0.24
	50–54	217 (97.7)	6 (2.69)	
	55–59	259 (95.2)	13 (4.8)	
	60–64	398 (93.0)	30 (7.0)	
	65–69	563 (94.5)	33 (5.5)	
	70–74	586 (95.6)	27 (4.4)	
	75–79	632 (95.0)	33 (5.0)	
	80–84	492 (94.6)	28 (5.4)	
	85+	415 (95.2)	16 (4.8)	
Cancer site	Colon	3488 (94.8)	191 (5.2)	*χ* ^2^ = 0.32 *p*‐value = 0.57
	Rectum	86 (93.5)	6 (6.5)	
Stage at diagnosis	Stage 1	882 (95.2)	45 (4.8)	*χ* ^2^ = 5.94 *p*‐value = 0.20
	Stage 2	1143 (94.2)	70 (5.8)	
	Stage 3	1016 (95.4)	49 (4.6)	
	Stage 4	252 (92.3)	21 (7.7)	
	Unknown	281 (95.9)	12 (4.1)	

Abbreviations: ARIA, Accessibility/Remoteness Index of Australia; EP, Emergency Presentation; SEIFA, Socioeconomic Index for Areas.

Out of six independent variables entered into logistic regressions (SEIFA quintiles, ARIA groups, age, gender, cancer site, and stage at diagnosis), only ARIA group (remoteness area) was significantly associated with EP within 30 days of hospital admission, with the inner region having higher odds of presenting to ED within 30 days of hospital admissions compared to major cities (Inner regional: AOR = 1.45) (Table 6).

**TABLE 6:** Factors associated with EPs within 30 days of hospital admissions.

Variables	Categories	EP within 30 days of hospital admissions	
		AOR (95% CI)	*p*
SEIFA Quintiles	Q 1	1.01 (0.62–1.66)	0.95
	Q 2	1.09 (0.66–1.79)	0.74
	Q 3	1.24 (0.76–2.03)	0.38
	Q 4	0.73 (0.42–1.27)	0.27
	Q 5	Reference	
ARIA groups	Major cities	Reference	
	Inner regional	1.45 (1.02–2.06)	0.04
	Outer regional/remote	1.20 (0.65–2.23)	0.56
Age (years)	45–59	0.93 (0.49–1.76)	0.83
	60–64	1.33 (0.70–2.51)	0.38
	65–69	1.04 (0.55–1.94)	0.91
	70–74	0.85 (0.45–1.62)	0.63
	75–79	0.94 (0.50–1.75)	0.84
	80–84	1.03 (0.54–1.95)	0.92
	85+	Reference	
Stage at diagnosis	Stage 1	Reference	
	Stage 2	1.25 (0.84–1.85)	0.28
	Stage 3	0.93 (0.60–1.43)	0.74
	Stage 4	1.67 (0.96–2.91)	0.07
	Unknown stage	0.76 (0.37–1.56)	0.46
Gender	Male	References	
	Female	0.85 (0.63–1.15)	0.29
Cancer site	Colon	Reference	
	Rectal	1.57 (0.63–3.90)	0.33

Abbreviations: AOR, adjusted odds ratio; ARIA, accessibility/remoteness index of Australia; CI, confidence interval; EP, emergency presentations; SEIFA, socioeconomic index for areas.

## 4 Discussion

This study provides insights into geographic and socioeconomic variations in CRC patients' EPs within 6 months before cancer diagnosis. Twenty‐one per cent of CRC patients had an EP for CRC‐related symptoms. Thirty‐four per cent of these occurred within 6 months before diagnosis. The highest rates were observed in 2019 (37%) and 2020 (38%). The increased rates during the COVID‐19 pandemic may be attributed to a decline in participation in the National Bowel Cancer Screening Program [43], which potentially leads to the increased proportions of patients presenting to the ED [44].

Significant disparities were observed among socioeconomically disadvantaged groups and those residing in regional and remote areas. Similar findings have been reported in other studies, highlighting socioeconomic disparities among patients with a diagnosis of CRC through EPs, even within systems that provide universal health care [4, 14, 45]. Several factors may contribute to these disparities. First, individuals from more disadvantaged backgrounds often face barriers to timely healthcare access, such as lower health literacy, limited transportation, and financial constraints, which can result in delayed healthcare‐seeking behaviours [46]. In Australia, despite substantial government subsidies, out‐of‐pocket expenses remain a significant part of health care costs, comprising around 15% of total health expenditures [47]. This financial burden creates barriers to access and intensifies health inequalities. The delays due to healthcare barriers might result in patients presenting to the ED when symptoms have become severe or advanced. Additionally, participation in preventive measures like cancer screening programs tends to be lower among socioeconomically disadvantaged populations, further contributing to the higher likelihood of emergency diagnoses [48].

Geographic accessibility to services also plays a crucial role in ED presentations. Patients from inner regional areas and those from outer regional and remote areas were more likely to present to the ED compared to those from major cities. This finding aligns with the study by Campbell et al. demonstrating that CRC patients residing in remote regions in the UK were more likely to present as emergency cases and had higher mortality compared to urban patients [49]. Rural residents may experience delayed diagnoses and treatment due to healthcare access barriers, which can exacerbate their conditions and increase the likelihood of EPs [50].

Geographic accessibility to services was significantly associated with EPs within 30 days of hospitalisation. Individuals from inner regional areas were more likely to visit an ED within 30 days following their hospital treatment compared to those from major cities. Evidence from other studies also demonstrates that rural populations are more likely to be readmitted. For example, research involving patients aged 65 years and older revealed that those in rural areas were more likely to experience 30‐day readmissions, possibly suggesting a lower quality of care in rural hospitals [51]. Similarly, a study in Nebraska, US, reported that rural residents were 40% less likely than their urban counterparts to undergo a laparoscopic colectomy, a surgical procedure known to enhance postoperative outcomes [52]. This disparity suggests that rural populations may face challenges in accessing advanced healthcare procedures, contributing to poorer health outcomes and a greater reliance on emergency services post‐hospitalisation. These findings highlight the need for improved healthcare access and support services in regional and rural areas to reduce emergency readmissions and improve the overall quality of care in these regions.

Age at diagnosis also influenced ED presentations, with older patients showing lower odds compared to those aged 45–49 years. Similarly, previous studies indicate that younger patients are more likely to be diagnosed through EPs and experience longer intervals between referral and diagnosis [53]. The misconception that CRC is primarily an older adult's disease can lead to delays in diagnosis among younger individuals, as both patients and healthcare providers may attribute symptoms to less severe conditions [54, 55]. This results in more advanced disease at the time of diagnosis, often requiring emergency care.

The stage of cancer at diagnosis significantly affected the likelihood of ED presentation. Advanced (stages 3–4) and unknown stages were associated with substantially higher odds of ED presentation compared to stage 1. Similar findings from other studies indicate that advanced cancer stages are associated with more severe symptoms and complications, resulting in EPs [56]. Early‐stage cancers are typically less symptomatic and can often be managed through scheduled outpatient care, whereas advanced‐stage cancers may present with more severe symptoms that require urgent attention [57]. These findings underscore the critical role of early cancer detection in mitigating avoidable ED admissions. For instance, Australia's NBCSP [6, 7], has demonstrated success in reducing advanced‐stage colorectal cancer (CRC) diagnoses and lowering ED presentation rates through the timely identification of precancerous lesions and localized tumours [8, 9]. Therefore, increasing participation in the NBCSP could enhance early cancer detection, potentially reducing late‐stage diagnoses and alleviating the burden on ED [7, 9].

The study analysed statewide data collected over 14 years, enhancing the representativeness of the findings for Victoria and their generalizability to other states and territories of Australia. However, the study had some limitations. First, variables such as screening status were not included in the datasets, which could be among the potential factors influencing EPs. Secondly, although EPs before cancer diagnosis can provide insights into patients' access to and utilisation of primary care and preventive services and suggestive of the diagnosis pathway, we were unable to determine whether the EPs we assessed directly led to a cancer diagnosis.

## 5 Conclusion

Despite a reduction in the number of EPs before CRC diagnosis in recent years in Victoria, significant disparities persist across socioeconomic groups, remoteness areas, and country of birth, even in the context of universal screening programs and healthcare. These findings underscore the need for targeted screening initiatives, enhanced primary care, and preventive services focused on lower socioeconomic and regional and remote populations with a higher risk of EPs. Moreover, integrating preventive measures, strengthening referral pathways, and improving rural healthcare quality can reduce avoidable hospital readmissions, offering additional cost savings. Future research should examine the economic benefits of reducing EP disparities and the impact on long‐term healthcare expenditures.

## Author Contributions

**Bedasa Taye Merga:** conceptualization, investigation, validation, methodology, formal analysis, data curation, writing – original draft, software. **Nikki McCaffrey:** conceptualization, writing – original draft, validation, methodology, supervision, writing – review and editing. **Suzanne Robinson:** conceptualization, validation, methodology, supervision, writing – original draft, writing – review and editing. **Craig Sinclair:** validation, writing – review and editing, methodology. **Justin M. Yeung:** validation, methodology, writing – review and editing. **Anita Lal:** conceptualization, methodology, validation, writing – original draft, writing – review and editing, supervision, resources.

## Ethics Statement

Approval from the Australian Institute for Health and Welfare Human Research Ethics Committee (reference no EO2022/3/1335 (ID 3743)) and Deakin University Human Research Ethics Committee (reference no. 2024/HE000327) was provided for this project. The Ethics Committee granted a waiver of written informed consent for this study.

## Conflicts of Interest

The authors declare no conflicts of interest.

## Data Availability

The data supporting this study's findings are available from the Victorian Department of Health and the Australian Institute of Health and Welfare, but restrictions apply. These data were used under licence for the current study and are not publicly available.
